# Patient-centered care in pulmonary fibrosis: access, anticipate, and act

**DOI:** 10.1186/s12931-024-02997-7

**Published:** 2024-11-01

**Authors:** Delian E. Hofman, Tonia Magrì, Catharina C. Moor, Luca Richeldi, Marlies S. Wijsenbeek, Yuko Waseda

**Affiliations:** 1 Department of Respiratory Medicine, Center of Excellence for Interstitial Lung Disease, Erasmus University Medical Center, Rotterdam, The Netherlands; 2 Università Cattolica del Sacro Cuore, Rome, Italy; 3 Division of Pulmonary Medicine, Fondazione Policlinico Universitario A. Gemelli IRCSS, Rome, Italy; 4 Department of Respiratory Medicine, Faculty of Medical Sciences, University of Fukui, Eiheiji, Japan

**Keywords:** Comprehensive care, Holistic care, Fibrotic interstitial lung diseases

## Abstract

Comprehensive care integrates individual patient needs and is highly valued for patients with pulmonary fibrosis (PF). The importance of a patient-centered care approach is rooted in the unpredictable progressiveness of the disease course in PF. The respiratory impairment associated with PF has a major impact on the quality of life for both patients and their caregivers. We believe that prioritizing patient preferences could improve the shared decision making process and may ultimately lead to better health outcomes. Despite the growing emphasis for this approach, it remains challenging to adopt it in clinical practice. In this review, we propose the comprehensive Triple A Care Model, consisting of the domains Access, Anticipate, and Act, which emphasizes core elements of patient-centered care for patients with PF. We will provide an overview of the unmet needs in care for patients with PF and elaborate on the current methods for delivering patient-centered care. The latest insights into symptom management and supportive measures and several approaches to improving access to care are discussed, in line with the most recent guidelines.

## Introduction

Interstitial lung disease (ILD) comprises a heterogeneous group of diseases affecting the lung parenchyma and is characterized by inflammation, pulmonary fibrosis (PF) or a combination of both [1, 2]. Idiopathic pulmonary fibrosis (IPF) is the most common type of PF, with a prevalence estimated to be in the range of 0.09–1.30 per 10,000 individuals globally [3]. It is characterized by inevitable progression in terms of worsening respiratory symptoms, a decline in lung function, and a decrease in health-related quality of life (HRQOL), eventually resulting in respiratory failure and early mortality. Regardless of the origin of the disease, PF is a life-altering condition due to its chronic and burdensome symptoms, including dyspnea, intractable cough, and disruptive fatigue [4]. Various forms of PF have an IPF-like disease progression, also known as progressive pulmonary fibrosis (PPF) [2]. For example, up to 58% of patients with hypersensitivity pneumonitis (HP), 51% of patients with unclassifiable ILD (U-ILD), and 45% of patients with connective tissue disease-associated interstitial lung disease (CTD-ILD) are at risk of developing PPF according to registry data [5]. Antifibrotic agents slow down the disease progression in IPF and PPF, but do not improve symptom relief or HRQOL [6–9]. Thus, better non-pharmacological and pharmacological treatment options aimed at symptom relief are crucial for patients with PF [7, 10]. The psychological burden of PF is substantial not only for patients but also for their caregivers [11–13], who frequently provide mental and physical support. Hence, clinicians should actively involve caregivers throughout the entire disease course [14].

The general healthcare paradigm is slowly shifting from a more paternalistic model toward a patient-centered and cooperative approach between the clinician and the patient. In this care model, the primary focus is to address the specific health needs, values, and preferences of patients and their caregivers. These aspects serve as the cornerstone for guiding many healthcare decisions in patient-centered care [15].

Patient-centered care in patients with PF encompasses many facets, each with its own set of nuances and potential barriers to integration. In this narrative review, the Triple A Care Model is introduced, integrating the domains of patient-centered care described in the published literature, while also addressing the unmet needs in providing comprehensive care.

## The Triple A Care Model for patient-centered care

**Fig. 1:**
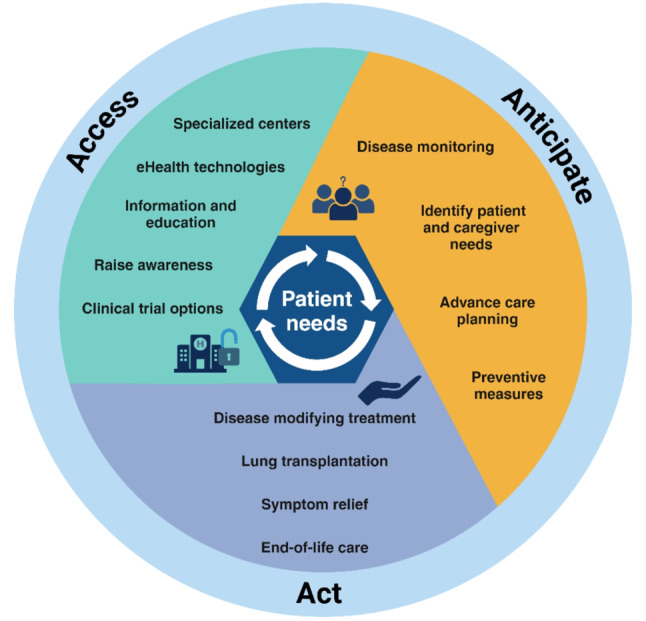
The Triple A Care Model for patient-centered care in pulmonary fibrosis: ‘Access, Anticipate, Act’

We propose the Triple A Care Model for patient-centered care in pulmonary fibrosis, ‘Access, Anticipate, and Act’ (Fig. 1). This model serves as a tool to deliver personalized care. We believe that by routinely identifying patient’s needs and evaluating whether the topics of the three domains of the model are targeted, PF care can be optimized. This model can help raise awareness of the importance of holistic care for patients with PF. “Access” refers to raising PF awareness among clinicians and the general public and adopting of eHealth technologies, thereby providing early access to specialized centers, information and education, and clinical trial options. “Anticipate” encompasses continuous assessment of patient needs, preventive measures, disease monitoring, and advance care planning (ACP). Finally, the “Act” domain focusses on the timely initiation of disease-modifying treatment and symptom relief, early evaluation for lung transplantation (LTx), and promptly adopting end-of-life care.

## Access

### Specialized centers

Patients with PF are usually diagnosed and treated in specialized centers, which are often geographically restricted [16]. This may lead to inequalities in healthcare, particularly affecting patients living further away from specialized centers [17, 18]. To ensure a timely and accurate diagnosis, multidisciplinary collaboration is needed [16, 19], integrating clinical, radiological, and pathological expertise [20].

### eHealth technologies

Distances to expert care can nowadays be bridged by using eHealth technologies [21]. The World Health Organization (WHO) developed the Global Strategy on Digital Health 2020–2025 [22], which advocates for digital health integration to strengthen health systems globally. For example, virtual multidisciplinary teams (MDTs) allow for the sharing of expertise from specialized centers. Furthermore, various online home monitoring programs enable remote patient monitoring, including home spirometry [23–25], pulse-oximetry, and digital patient-reported outcome measures (PROMs) [26]. This may reduce cumbersome travel to the hospital and promote patient self-management while allowing for more frequent monitoring [25, 27, 28]. While home monitoring offers many benefits, substantial challenges must be addressed. There is a risk of increasing health disparities, particularly for patients with low digital health literacy or those who are socioeconomically disadvantaged [28]. Furthermore, quality control of home-based measurements should be ensured [28].

### Information and education

In order to make realistic choices about disease management and priorities in living with the disease, patients need to be well informed [29–32]. Currently, no structured educational program for patients with PF exists. Educational resources, such as YouTube and social media [33], which are publicly available, frequently provide incomplete and inaccurate information [34, 35]. Improving the quality and reliability of the information provided to patients with PF is necessary. Key patient-reported educational topics in qualitative interviews include comprehensive disease education, symptom management, clinical tests (i.e., lung function tests), self-sufficiency, oxygen use, pharmacological treatment, and end-of-life counseling [29]. Moreover, as patients adopt other coping strategies at different stages of disease progression, information and education should be tailored to the disease stage [14]. Specialized centers and patient organizations both play an important role in raising awareness by advocating for better care and spreading accurate disease information [36]. Collaboration between clinicians and patient organizations is needed to provide reliable information in layperson’s terms.

### Raise awareness

Besides patients that need education, there is also a lack of widespread awareness among respiratory clinicians and primary care physicians [37], often resulting in a delay in accurately diagnosing PF and referral to specialized centers [16, 19, 30, 38–40]. These delays are reported in various studies with medians ranging from 7 months [19] to 2 years [39] and vary significantly among individuals [30]. There is evidence that patient outcomes can improve with earlier diagnosis. Early diagnosis may lead to early initiation of disease modifying therapy, options to provide symptom relief, early evaluation for LTx, and prolonged patient survival [41]. Moreover, a long diagnostic trajectory leads to uncertainty and distress for patients and their relatives [30]. Specialized centers and patient organizations contribute to spreading awareness about PF, for example, by organizing the global PF Awareness month and sharing information about PF through social media channels and online platforms [42]. Disseminating accurate information not only among the community of ILD experts, but also to primary care physicians and governmental parties, is important to improve access to care and treatments around the world.

### Clinical trial options

Although trials on new treatment options are crucial to improve outcomes for patients, many patients are not informed about the opportunity to participate in clinical trials, despite the recommendation in the IPF and PPF guidelines [2]. The use of digital health technologies could improve the accessibility of enrollment to clinical trials by reducing the burden of visits typical of conventional trials and at the same time improve the quantity of data collection [43].

## Anticipate

### Disease monitoring

Close monitoring of patients with PF is crucial for the timely identification and management of progressive disease as well as for the initiation of supportive measures. Monitoring should include regular assessments of symptoms, physiological parameters (pulmonary function and 6-minute walk test (6-MWT)), and when necessary, high-resolution computed tomography (HRCT) [44]. International guidelines recommend that patients with IPF and PPF have their forced vital capacity (FVC) and diffusing capacity of the lungs for carbon monoxide (DLCO) measured every 3 to 6 months, or more frequently if clinically necessary [2]. Furthermore, regularly asking for medication side-effects and symptoms is relevant to timely adjust therapy.

### Identify patient and caregiver needs

Optimizing patient-centered care is not possible without understanding the needs of patients and their caregivers. Patient needs should be the central focus and anticipated at every stage of care, as displayed in Fig. 1. In the last decade, various studies have sought to identify key needs through surveys and interviews with patients and their partners [45]. The key identified needs are increasing disease awareness and disease-specific education, early and accurate diagnosis, access to specialized centers, access to antifibrotic treatment, a focus on symptom-based management, clinical trial access, and psychological support [12, 46–50]. In daily practice, extensive needs assessment may be pressured by time constraints. PROMs administered in advance of the consultation could be used to assess structural disease impact and patient well-being by self-reporting health status [51, 52] and symptom burden, and could help to identify and monitor unmet supportive needs. Complementary to PROMs, patient reported experience measures (PREMs) are tools used to measure patient’s healthcare experiences and could help to improve care [53]. Furthermore, caregivers also have unmet needs [13] and should be supported with proper strategies, including educational programs and social and psychological support [54, 55].

### Advance care planning

Palliative care aims to improve HRQOL for patients and their caregivers throughout the disease course, which is particularly relevant in PF due to the high disease burden and often progressive disease course. As many people interpret palliative care as end-of-life care [56], careful explanation or the use of alternative wording, such as supportive care, may be used to avoid causing unnecessary worries in patients and families. ACP plays a pivotal role in palliative care. It encompasses the ongoing communication process to assess the goals and preferences of patients and caregivers regarding future medical care, taking into account the religion, culture, and all other beliefs [57]. Initiating ACP is often perceived as a sensitive and complex topic, not in the least because prognostication for individual patients with different forms of PF remains challenging. Other obstacles include time constraints, personal beliefs, and lack of training [58]. Healthcare professionals should be offered training to develop effective and empathetic communication skills [59]. Furthermore, collaborating with palliative care specialists, where possible, could enhance a holistic approach to palliative care. Acute exacerbations frequently occur, forcing patients, their families, and healthcare providers to make urgent decisions regarding issues as intensive care unit (ICU) admission and mechanical ventilation. Timely discussion of treatment limitations, in terms of resuscitation and (bridgeable) ICU hospitalization, is important for preventing unwanted medical interventions. Given that ICU hospitalization is associated with increased mortality in all forms of PF [60], patients need to be informed about these possible negative consequences to make well-thought decisions.

### Preventive measures

Although there are few studies on options to prevent acute worsening of the disease in patients with PF, several preventive healthcare measures must be considered. When elective, mechanical ventilation should be avoided where possible as it may trigger worsening of PF. Furthermore, vaccinations for respiratory disease against COVID-19, influenza, and pneumococcal infections are important in patients with PF to prevent infections that could provoke acute exacerbations and are recommended by the Center for Disease Control and Prevention (CDC) [61]. Cigarette smoking is believed to be a risk factor for the development of IPF [62]. Smoking cessation is integral to PF management as smoking is associated with progressive worsening and increases the risk for lung cancer [63].

### ACT

For each individual patient, treatment benefits, drawbacks, and patient preferences should be considered. One of the challenges in care for patients with PF is the difficulty in predicting disease behavior and response to therapy in the individual patient [64]. Reimbursement strategies of care are variable and, therefore, access to treatments and supportive measures may vary around the world. This section of the review summarizes current and emerging options for disease-modifying treatment, LTx, supportive care, and end-of-life care based on the most recent literature and guidelines.

### Disease-modifying treatment

#### Antifibrotic therapy

International guidelines recommend the use of the antifibrotic agents pirfenidone for IPF and nintedanib for both IPF and PPF [2]. Specific criteria are defined for PPF [2], with at least two out of three of the following: worsening symptoms, radiological progression, and physiological progression [65], occurring within the past year [66, 67]. Antifibrotic therapy has been demonstrated to be effective in reducing the decline in lung capacity in patients with IPF [6, 7], systemic sclerosis-associated ILD (SSc-ILD) [8], and PPF [68], irrespective of the underlying ILD diagnosis [69]. Long-term registry studies and post-hoc analyses of clinical trials also suggest that antifibrotic therapy reduces the risk of death and acute exacerbations in patients with IPF [6, 7, 70, 71]. Randomized controlled trials in patients with IPF and PPF did not show an effect of antifibrotic therapy on improving symptoms or HRQOL [6–9]. A post-hoc analysis of the INBUILD study demonstrated that nintedanib may slow down the worsening of cough, dyspnea, and fatigue in patients with PPF, as assessed with the living with pulmonary fibrosis (L-PF) questionnaire [72]. The differences in outcomes between the INBUILD and previous studies may be attributable to the questionnaire used, with the L-PF potentially being more sensitive to changes in symptoms within this population compared to questionnaires used in previous studies. Additionally, a small observational study found that pirfenidone improved the 24-hour cough count and cough-related HRQOL in IPF patients [73]. Antifibrotic therapy can cause disabling side effects, such as photosensitivity and nausea in patients treated with pirfenidone and diarrhea in patients treated with nintedanib. Nevertheless, side effects are generally manageable, and overall, patients are fairly satisfied with antifibrotic therapy [74]. It is imperative to manage side effects to the greatest extent possible to optimize HRQOL. Currently, various other antifibrotic agents for IPF and PPF are being investigated and may be used in the future [75, 76].

#### Anti-inflammatory therapy

Anti-inflammatory therapy is usually the first-line treatment in inflammation-driven ILDs, e.g., CTD-ILD, sarcoidosis, and idiopathic inflammatory myopathy. However, despite its wide use, evidence-based support from clinical trials remains scarce, except for that of patients with SSc-ILD [77–79]. Moreover, anti-inflammatory therapy may also lead to adverse effects and is associated with an increased risk of infections. As many indications are off-label, benefits and drawbacks should be considered in consultation with the patient. In the management of IPF, anti-inflammatory therapy is not recommended given the increased risk of mortality and adverse effects without clear benefit [1, 80]. It is conceivable that similar risks may exist in patients with a usual interstitial pneumonia (UIP) pattern in conditions other than IPF. Recently, it was shown that the use of anti-inflammatory therapy in patients with short telomeres was associated with reduced survival [81]. Except for SSc-ILD [82–84], it remains unclear if combining antifibrotic treatment with anti-inflammatory therapy is beneficial for the treatment of PPF.

### Lung transplantation

LTx is currently the final treatment option for patients with advanced PF. LTx can extend life expectancy and enhance HRQOL [85]. The International Society for Heart and Lung Transplantation (ISHLT) emphasizes the importance of early referral to a transplant center for LTx evaluation due to the potential for rapid progression of PF [86]. Furthermore, the ISHLT strongly recommends LTx in carefully selected patients [86]. In patients with familial pulmonary fibrosis or fibrosis in CTD-ILD, early discussion with a transplant center is recommended, as screening may take more time because of the multisystem involvement of the disease.

### Symptom relief

**Fig. 2:**
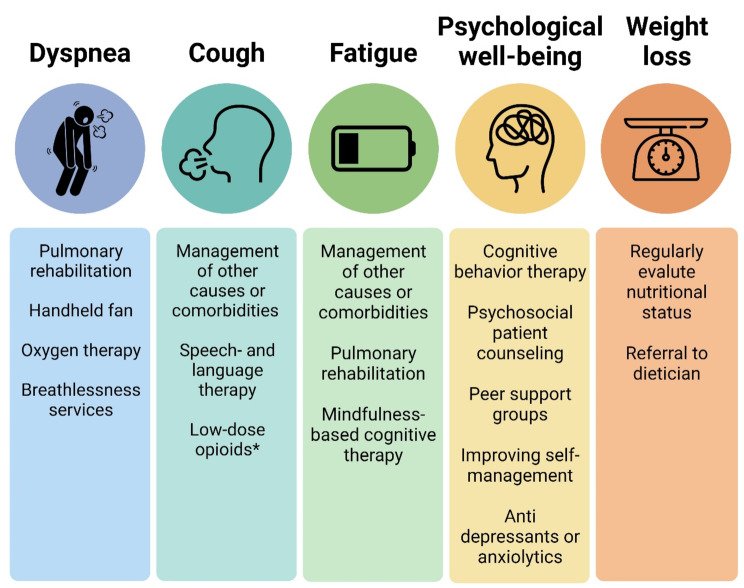
Commonly used options to relief symptoms in patients with pulmonary fibrosis

The principal symptoms of PF that profoundly impact patient HRQOL include dyspnea, cough, and fatigue. Managing these symptoms with currently available treatments remains challenging [87]. Figure 2 illustrates the commonly used options to relief symptoms in patients with PF.

#### Dyspnea

Dyspnea is the prevailing symptom in most patients with PF, with a prevalence of 90% at diagnosis [88]. It leads to decreased exercise capacity and diminished HRQOL and is associated with increased anxiety [89–92]. In the more advanced stages of disease, dyspnea might persist even in a resting state. Dyspnea is a predictor of poor survival in patients with IPF [93, 94]. Managing dyspnea is complex, and treatment primarily involves non-pharmacological interventions [95].

Long-term oxygen therapy (LTOT) is recommended by international guidelines for patients with ILD and severe chronic resting hypoxemia [96, 97]. However, there is limited evidence regarding the timing [98–100], long-term effects on the disease trajectory, and impact on symptoms [101]. The more recent guidelines on symptom management for adults with serious respiratory illness suggest a personalized approach through shared decision making for administering supplemental oxygen to reduce symptoms [95]. Ambulatory oxygen use is suggested for patients with ILD and severe exertional hypoxemia [96]. The AmbOx randomized controlled trial demonstrated a favorable effect of ambulatory oxygen versus placebo air on HRQOL in patients with ILD and isolated exertional hypoxia [102]. Conversely, a Cochrane and systematic review showed no significant effect of ambulatory oxygen use on dyspnea scores in patients with IPF, mainly during exercise tests, although exercise capacity improved [103]. However, the results may not be generalizable due to the low quality of the reviewed studies, which included uncontrolled randomized controlled trials with small sample sizes. The use of supplementary oxygen may pose challenges to patients, leading to subsequent drawbacks in oxygen use. They may find it difficult to embrace oxygen therapy due to social stigma and the cumbersome equipment of oxygen, which can impair their independence [104–107].

Recently published European Respiratory Society (ERS) guidelines suggest the use of graded exercise therapy and multicomponent breathlessness services that offer more than one intervention to reduce symptoms [95]. Two controlled trials demonstrated positive long-term effects of pulmonary rehabilitation on exercise tolerance in patients with various ILDs [108, 109]. Additionally, using easily accessible hand-held fans that blow air on a patient’s face may help reduce the sensation of breathlessness, offering potential benefits for patients with PF [95, 110–112].

Evidence-based pharmacological management of dyspnea is lacking [113]. Opioids and benzodiazepines may alleviate dyspnea by inhibiting respiratory drive and modulating cortical activity [113]. ERS guidelines do not recommend the use of opioids for the treatment of dyspnea in patients with a serious respiratory illness, including PF [95]. One randomized controlled trial comparing the effect of low-dose oral morphine to a placebo in patients with PF found no significant effect on dyspnea scores for short-term use [114]. In fact, patients in the treatment group had an increased risk of adverse effects such as constipation, nausea, and confusion. A more recent randomized controlled trial showed no effect of several morphine dosages on breathlessness and morphine was associated with more harm [115]. The certainty of evidence of these results is low due to the small sample size of this study. On the other hand, opioids and benzodiazepines appear to be safe as they do not seem to increase the risk of hospital admissions or mortality [116]. In earlier disease stages, clinicians are often hesitant to prescribe opioids due to the potential risk of addiction. Opioids are more frequently prescribed in end-of-life stages; however, no studies have been performed on the effect of opioid use in these advanced disease stages.

#### Cough

Cough is commonly reported, affecting up to 80% of patients with PF [88]. Cough accounts for a substantial disease burden [117] and has many negative impacts on patients, including raucous voice, impaired sleep, musculoskeletal pain of the chest, social isolation, and urine incontinence [118]. The severity of cough is independently associated with diminished HRQOL, a greater annualized decline in DLCO, and reduced transplant-free survival in patients with PF [119]. The pathophysiology of cough is complex and involves mechanical, biochemical, and neurosensory factors. However, not all mechanisms are completely understood [120–122].

The first step in the management of cough is to evaluate and treat other possible causes of cough, such as gastroesophageal reflux disease (GERD) and obstructive sleep apnea (OSA) [118]. Among non-pharmacological methods, speech and language therapy seems to be effective for patients with refractory chronic cough [123–125], however, its impact on patients with PF requires further investigation [126]. Many pharmaceutical options to reduce cough in PF are being investigated. Two recent randomized controlled trials showed that low-dose morphine reduced cough frequency compared to placebo in patients with IPF, suggesting that low dose opioids may be a treatment option when cough is debilitating [127, 128]. Further larger trials are currently ongoing [NCT05964335]. A trial of low-dose slow-release morphine is also recommended by ERS guidelines for treating patients with chronic refractory cough [129]. Although there is limited evidence, gabapentin may be considered for treating ILD-associated cough, following the CHEST guidelines [126]. One randomized controlled trial investigating the effects of gabapentin for patients with ILD is ongoing [ChiCTR2100045202]. Other neuromodulators that are being explored for their ability to reduce cough in patients with PF are amitriptyline and duloxetine [NCT05120934]. In an older and small randomized controlled trial, thalidomide showed positive effects on cough-specific quality of life in patients with IPF-related cough [130]. Given the lack of further studies confirming its efficacy and potential severe side effects, thalidomide is not recommended in guidelines [95, 126, 129]. Finally, in one small randomized controlled trial, suppression of the cough reflex by the P2X3 inhibitor gefapixant in patients with IPF did not significantly improve IPF-related cough [131]. However, this study had certain methodological limitations. Studies with other P2X3 receptor antagonists in IPF populations are ongoing [NCT05185089].

#### Fatigue

The prevalence of fatigue varies among different ILDs, with an estimated prevalence of up to 95% in patients with IPF, 90% in patients with sarcoidosis, 87% in patients with HP, and 71% in patients with other non-IPF ILDs [132]. It has a major impact on HRQOL but is difficult to manage and often underrecognized [132]. The etiology of fatigue is multifactorial and likely not uniform across different forms of ILD. For instance, sarcoidosis-associated fatigue is potentially driven by several factors including disease-related inflammation and small-fiber neuropathy, but is often not associated with lung function tests and exercise capacity [133–136]. In contrast, fatigue severity in patients with IPF seems to be associated with lung function and diffusion impairment [137]. Moreover, concomitant comorbidities, such as sleep disorders, diabetes, anemia, anxiety, and depression, as well as pharmaceutical agents used in PF, such as pirfenidone and corticosteroids, may also cause or worsen fatigue [132]. For this reason, it is important to adopt a comprehensive approach for the treatment of fatigue. Validated PROMs may be used to structurally assess the presence and severity of fatigue [138]. Several pharmacological and non-pharmacological treatment options can be considered. Pulmonary rehabilitation may improve fatigue in patients with ILD [139, 140]. Recently, a randomized controlled trial in 99 patients with sarcoidosis, including patients with fibrotic sarcoidosis, indicated that online mindfulness-based cognitive therapy improved fatigue [141]. Three exploratory randomized controlled trials of the effects of neurostimulants (dexmethylphenidate hydrochloride [142], armodafinil [143], and methylphenidate [144]) on sarcoidosis-related fatigue showed promising results, but these agents have not been investigated for the treatment of fatigue in other ILDs.

#### Psychosocial well-being

Anxiety and depression are common among patients with PF, with the prevalence of depression estimated to be between 14% and 49% and that of anxiety estimated to be between 21% and 60% [145]. The prevalence of these conditions may be underestimated due to the lack of reporting and questioning of these symptoms. One of the numerous factors contributing to anxiety and depression is the burden of living with a chronic and often progressive disease that is characterized by an unpredictable disease course. Moreover, factors that can worsen psychological well-being, and increase the risk of social isolation, include inability to work, loss of independence in daily activities, feeling like a burden to caregivers, and dependence on supplemental oxygen. Nonetheless, many patients with PF do not receive any form of treatment for their depressive symptoms [146]. Non-pharmacological strategies for managing psychological impairment include patient counseling, cognitive behavioral therapy, and peer support group participation [54, 55]. Psychologists and PF specialist nurses can contribute to alleviating psychological distress by educating patients on how to cope with anxiety and depression, and providing emotional and practical support. To our knowledge, no studies have investigated the effects of antidepressants on psychological distress in patients with PF. Online home monitoring programs may support in the enhancement of psychosocial well-being, potentially by improving self-management, early detection of disease deterioration, and low-threshold communication with the hospital [23].

#### Nutritional status and weight loss

When adopting a holistic approach to patient care, it is important to assess general physical status with a particular focus on nutritional status, for example through simple tools such as the Mini Nutritional Assessment (MNA). Several factors negatively impact nutritional status in patients with ILD, including ageing, respiratory muscle loss, systemic inflammation, hypercatabolism, a sedentary lifestyle, an inappropriate diet, and pharmacological therapy [147]. Body weight loss is independently associated with the survival of patients with IPF, and a decrease in BMI of more than 5% per year is associated with increased mortality, regardless of FVC decline [148, 149]. However, it remains unclear whether optimizing weight could lead to better outcomes in patients with PF.

#### Management of comorbidities

PF often affects the elderly population and is associated with a wide range of comorbidities, which can affect survival, prognosis, and HRQOL [150]. The most common comorbidities associated with PF include GERD, OSA, pulmonary hypertension (PH), ischemic heart disease, lung cancer, and chronic obstructive pulmonary disease (COPD)/emphysema [151]. Among these comorbidities, cardiovascular disease and lung cancer are the two comorbidities with the greatest impact on mortality [152].

The therapeutic approach for treating comorbidities is often challenging. For the management of PH in patients with ILD, several clinical trials investigating drugs generally used for primary PH showed no improvement in patients with ILDs [153–158]. According to the 2022 European Society of Cardiology (ESC)/ERS guidelines [159], the therapeutic approach for ILD-associated PH starts with optimizing the treatment of the underlying disease. Routine use of other PH medications is not recommended for patients with ILD [159]. According to the results of the INCREASE randomized controlled trial [160], inhaled treprostinil has been approved by the Food and Drug Administration (FDA) for the treatment of patients with PH-associated ILD. However, it has not yet received approval from the European Medicines Agency (EMA). Furthermore, early referral for LTx evaluation is recommended given the significant impact of ILD-associated PH [86]. Patients with IPF, and potentially other forms of PF, are considered to have a greater risk of concomitant lung cancer, likely due to shared pathogenic pathways [161]. The prevalence of lung cancer among patients with IPF varies from approximately 7–31% [162]. The therapeutic approach remains challenging with limited data and no consensus for the treatment of patients with both PF and lung cancer [17, 163]. To develop a personalized and tailored management plan, decisions on therapeutic approaches should be carefully elaborated with the patient, involving a multidisciplinary team of healthcare providers.

*Low-dose opioids seem to alleviate cough, but there are no current guidelines recommending their use for pulmonary fibrosis. Further studies investigating their effectiveness are ongoing. Potential side effects of opioids should be considered.

### End-of-life care

Although many patients with IPF have a worse prognosis than those with lung cancer, end-of-life care is far less developed in this area, with less symptom relief achieved in IPF [164]. An observational study in dying patients with ILD compared to patients dying with lung cancer showed that patients with ILD had lower quality of dying and death and a lower frequency of participation in end-of-life discussions [165]. Information needs reported by patients regarding end-of-life care included knowledge of how death would happen to them and whether they would suffer [29]. Previous studies have shown that most patients prefer to die at home [166, 167]. A retrospective study demonstrated that dying at home or in a hospice seems to be feasible for patients with ILD when adopting early palliative care [168]. Timely conversations about the place of dying will allow patients to die surrounded by their family and in their chosen setting [45].

## Conclusion

Many steps forward have been made toward patient-centered care in the field of PF management. The proposed Triple A Care Model (Access, Anticipate, and Act) embraces important themes of patient-centered care in PF, including information and education, access to specialized care, addressing patient and caregiver needs, advance care planning, symptom relief, and end-of-life care. In conclusion, we believe that routinely addressing the domains of the Triple A Care model in clinical practice can improve the adoption of a more individually tailored approach to PF care.

## Data Availability

No datasets were generated or analysed during the current study.

## Abbreviations

ACP: Advance care planning
CDC: Center for Disease Control and Prevention
COPD: Chronic obstructive pulmonary disease
CTD-ILD: Connective tissue disease-associated interstitial lung disease
DLCO: Diffusing capacity of the lungs for carbon monoxide
EMA: European Medicines Agency
ERS: European Respiratory Society
ESC: European Society of Cardiology
FDA: Food and Drug Administration
FVC: Forced vital capacity
GERD: Gastroesophageal reflux disease
HP: Hypersensitivity pneumonitis
HRCT: High-resolution computed tomography
HRQOL: Health-related quality of life
ICU: Intensive care unit
ILD: Interstitial lung disease
IPF: Idiopathic pulmonary fibrosis
ISHLT: International Society for Heart and Lung Transplantation
L-PF: Living with pulmonary fibrosis
LTOT: Long-term oxygen therapy
LTx: Lung transplantation
MDT: Multidisciplinary team
MNA: Mini nutritional assessment
OSA: Obstructive sleep apnea
PF: Pulmonary fibrosis
PH: Pulmonary hypertension
PPF: Progressive pulmonary fibrosis
PREMs: Patient-reported experience measures
PROMs: Patient-reported outcome measures
SSc-ILD: Systemic sclerosis-associated ILD
U-ILD: Unclassifiable interstitial lung disease
UIP: Usual interstitial pneumonia
WHO: World Health Organization
6-MWT: 6-minute walk test
